# Associations between the spatiotemporal distribution of Kawasaki disease and environmental factors: evidence supporting a multifactorial etiologic model

**DOI:** 10.1038/s41598-021-93089-9

**Published:** 2021-07-16

**Authors:** Tisiana Low, Brian W. McCrindle, Brigitte Mueller, Chun-Po S. Fan, Emily Somerset, Sunita O’Shea, Leonard J. S. Tsuji, Hong Chen, Cedric Manlhiot

**Affiliations:** 1 Division of Cardiology, Department of Pediatrics, University of Toronto, Labatt Family Heart Centre, The Hospital for Sick Children, Toronto, ON Canada; 2 Department of Physical & Environmental Sciences, University of Toronto, Toronto, ON Canada; 3 Environmental Health Science and Research Bureau, Health Canada, Ottawa, ON Canada; 4 Present Address: Division of Cardiology, Department of Pediatrics, Johns Hopkins School of Medicine, Johns Hopkins University, 600 N. Wolfe Street, 1389 Blalock, Baltimore, MD 21287 USA

**Keywords:** Epidemiology, Paediatric research

## Abstract

The etiology of Kawasaki Disease (KD), the most common cause of acquired heart disease in children in developed countries, remains elusive, but could be multifactorial in nature as suggested by the numerous environmental and infectious exposures that have previously been linked to its epidemiology. There is still a lack of a comprehensive model describing these complex associations. We present a Bayesian disease model that provides insight in the spatiotemporal distribution of KD in Canada from 2004 to 2017. The disease model including environmental factors had improved Watanabe-Akaike information criterion (WAIC) compared to the base model which included only spatiotemporal and demographic effects and had excellent performance in recapitulating the spatiotemporal distribution of KD in Canada (98% and 86% spatial and temporal correlations, respectively). The model suggests an association between the distribution of KD and population composition, weather-related factors, aeroallergen exposure, pollution, atmospheric concentration of spores and algae, and the incidence of healthcare encounters for bacterial pneumonia or viral intestinal infections. This model could be the basis of a hypothetical data-driven framework for the spatiotemporal distribution of KD. It also generates novel hypotheses about the etiology of KD, and provides a basis for the future development of a predictive and surveillance model.

## Introduction

Kawasaki disease (KD) is an acute vasculitis of childhood which can be complicated by the development of coronary artery aneurysms (CAA)^1^. It is the most common cause of acquired heart disease in children in developed countries^1^, leading to important lifelong morbidity and mortality^2^. KD has been reported worldwide, with a marked geographic variation in incidence, and is characterized by a male predominance^3,4^. Despite extensive previous research, the etiology of the disease remains unknown. A genetic component is suggested from global epidemiological data, the distribution of the disease amongst ethnic groups, the clustering of cases within families and the increased risk of recurrence^5–10^. In addition, whole genome association studies have identified multiple genetic polymorphisms in inflammatory pathways which may increase the risk of KD^11,12^. Previous epidemiological studies have described associations between KD and various infectious diseases, pollen, pollution, early childhood environment and seasonality, albeit largely without considering the potential interaction between those factors^13–16^. Spatiotemporal clusters have been described in the distribution of KD and have been associated with local environmental factors and outbreaks of infectious diseases^15,17–19^. The prevailing consensus regarding the etiology of KD is that it occurs in individuals with a genetic and potential early childhood susceptibility, which predisposes to the development of a hyper-reactive immune response when exposed to an unidentified environmental or infectious trigger(s)^20^. In a recent studies, it was demonstrated that lower habitual exposure to environmental allergens and a preceding non-specific infectious exposure were associated with an increased risk of developing KD^21^. We herein present an integrative model that describes the variation in incidence of KD over time and space in Canada and its association with incidence of infections and allergic conditions, weather components, pollution, atmospheric biological particles and population composition.

## Methods

### KD data and identification of KD cases across Canada

Hospital admissions for Kawasaki disease were identified based on a primary or secondary discharge diagnosis of mucocutaneous lymph node syndrome (ICD-10-CA standard code M30.3). De-identified data from all hospital admissions (ages 0–18) from April 1, 2004 to March 31, 2017 were obtained from the Discharge Abstract Database (DAD) maintained by the Canadian Institute for Health Information (CIHI). Submission of administrative data to CIHI is legally mandated for all hospitals in Canada except for those in Quebec. In Canada, the entire population is covered by a single-payer universal health plan. A universal provincial health number was provided in an encrypted format for each case, which allowed us to exclude multiple admissions for a given patient. A previously validated algorithm was used to exclude all readmissions and transfers between hospitals^22^. For each KD case reported in DAD, the date of hospital admission and the forward sortation area (FSA)^23^ for the patient residence were provided. These data were previously used to determine and report the epidemiology of KD in Canada^22^.

### Population at risk, ethnicity, age distribution, population density and rural effect

Information on population, ethnicity, medium household income and age distribution was obtained from the Canadian Census 2006^24–26^, 2011^27^ and 2016^28^, and the National Household Survey^29^. Values from the 2006 census were used for 2004–2008, from the 2011 census/household survey for 2009–2014, and from the 2016 census for 2015–2017. FSAs without a Census population count were not considered in the analysis. Population density was calculated based on the land area file that contains the FSAs codes and sizes in square-km, derived from the FSA cartographic boundary file available through the University of Toronto Libraries^30^.

Ethnicity data in the Canadian Census is self-reported, based on the individual’s perception of their ethnic ancestry and respondents are allowed to select multiple ethnicity^31^. Given the strong predilection of KD for children of East Asian ethnicity, we considered the percentage of the population who self-reported Chinese, Japanese, Koreans and Taiwanese as one of their ethnicity. Finally, the percentages of the population in each FSA who live in postal codes classified as “rural” was calculated, with rural being defined as FSAs with a zero in the second digit.

### Healthcare encounters associated with infectious diseases and allergies

Population-level incidences of health care encounters associated with specific infections or allergy-related illnesses were obtained from both DAD and the voluntary National Ambulatory Care Reporting System Metadata (NACRS), which is also maintained by CIHI. Participation in NACRS is determined at the provincial level and achieves complete capture of ambulatory care in participating provinces. As it was not mandatory for all provinces to submit data to NACRS, models containing healthcare encounters for infections or atopic diseases as covariates were adjusted for the percentage of mandatory submission of infection and atopy data for each respective region. Data were obtained for all healthcare encounters across Canada which were associated with an ICD-10 diagnostic code for selected infections and allergy-related illnesses (see Table 1) in hospital admissions, and outpatient and community-based clinic encounters. These data were used to calculate monthly counts for codes and groups of codes. Infectious diseases and organisms included in this analysis were selected based on their prevalence in Canadian children. Infectious diseases were grouped in 2 separate ways: first, according to the type of etiologic agent (viral, bacterial, fungal infections or infections of unknown origins) and the affected physiological system (upper respiratory tract, lower respiratory tract, gastrointestinal tract and skin) and second, by the specific pathogen involved in the healthcare encounter. This dual system allowed us to consider both the contribution of specific pathogens but also of ailments for which clinical care rarely needs the identification of the specific pathogen and as such the majority of such encounters remain of indeterminate cause (e.g. viral intestinal infections).

**Table 1:** Comparison of hierarchical models with different temporal structures, adjustment factors and covariates.

Model/predictor (definition)	WAIC	Univariable SIR percentile					Multivariable SIR percentile				
		2.5th	25th	50th	75th	97.5th	2.5th	25th	50th	75th	97.5th
**Base model**	**23,134.0**										
Linear time	23,210.8										
Linear time + random walk	23,166.9										
Linear time + random walk + seasonality	23,134.0										
**Adjusted base model**	**23,128.0**										
Percentage population 0–4 years old per region	23,133.1	1.008	1.046	1.068	1.089	1.131	1.007	1.042	1.061	1.080	1.117
Percentage population self-identifying as East Asian per region	23,127.0	1.119	1.161	1.184	1.207	1.252	1.089	1.131	1.153	1.176	1.221
**Main regression model**	**23,059.7**										
**Exposures at the FSA level**											
Median household income in FSA^†^	23,125.5	0.882	0.912	0.929	0.946	0.978	0.900	0.931	0.947	0.964	0.997
Proportion of rural postal codes in FSA	23,127.5	0.864	0.894	0.910	0.926	0.957	0.898	0.930	0.947	0.964	0.998
Population density in FSA	23,128.3	1.001	1.049	1.075	1.102	1.154					
Weather											
Evaporation (m)^†^	23,124.7	1.021	1.068	1.093	1.119	1.170					
Soil temperature at 0–7 cm depth (layer 1, K)^†^	23,125.2	0.812	0.861	0.888	0.916	0.972					
Planetary boundary layer height, lowest part of the atmosphere (m)^†^	23,125.6	1.000	1.024	1.037	1.050	1.075					
Instantaneous eastward turbulent surface stress (N m^-2^)^†^	23,126.8	0.994	1.016	1.027	1.039	1.062					
Surface pressure (Pa)	23,127.3	0.977	1.035	1.067	1.100	1.164					
10 m U (zonal, eastward) wind component at 10 m (m s^−1^)	23,127.9	0.982	1.014	1.032	1.050	1.085					
2 m temperature (K)	23,128.1	0.846	0.897	0.925	0.954	1.011					
Total cloud cover (0–1)	23,128.1	0.929	0.958	0.973	0.989	1.018					
Total precipitation (m)	23,128.3	0.950	0.971	0.982	0.994	1.015					
10 m V (meridional, northward) wind component at 10 m (m s^−1^)	23,128.4	0.989	1.009	1.019	1.030	1.051					
Low vegetation cover (0–1)	23,128.7	0.893	0.950	0.981	1.013	1.077					
High vegetation cover (0–1)	23,129.0	0.936	0.978	1.001	1.024	1.071					
Photosynthetically active radiation (J m^−2^)	23,129.0	0.811	0.896	0.944	0.995	1.098					
Instantaneous northward turbulent surface stress (N m^−2^)	23,129.0	0.980	1.002	1.014	1.027	1.050					
Volumetric soil water layer at 0–7 cm depth (layer 1, m^3^ m^−3^)	23,129.2	0.953	0.981	0.997	1.012	1.043					
UV-radiation (J m)	23,129.2	0.828	0.908	0.953	1.001	1.097					
10 m wind speed at 10 m (m s^−1^)	23,129.6	0.963	0.993	1.010	1.027	1.059					
**Pollution (total column)**											
Sulphur dioxide and nitrogen oxide (SO_2 +_ NO_2_) (kg m^−2^)^†^	23,124.2	1.014	1.055	1.077	1.099	1.143	1.055	1.102	1.127	1.153	1.204
Ozone (kg m^−2^)	23,128.9	0.969	1.011	1.034	1.057	1.103					
Carbon monoxide (CO) (kg m^−2^)	23,129.5	0.949	0.993	1.017	1.041	1.088					
Fine particulate matter < 2.5 ug (kg m^−2^)	23,131.1	0.958	0.984	0.997	1.010	1.034					
**Spores**											
Algae^†,‡^	23,121.3	1.017	1.040	1.052	1.063	1.085	1.018	1.042	1.054	1.066	1.088
Deuteromycetes (fungi imperfecti)^†^	23,125.7	0.994	1.030	1.049	1.068	1.105	1.019	1.058	1.078	1.099	1.139
Myxomycetes^†^	23,126.5	0.993	1.018	1.030	1.043	1.065	0.989	1.014	1.027	1.040	1.063
Dothideomycetes^†^	23,126.8	0.905	0.937	0.954	0.971	1.004	0.892	0.925	0.943	0.961	0.996
Ascomycetes (other than dothideomycetes)	23,128.1	0.998	1.021	1.032	1.044	1.065					
Basidiomycetes	23,130.0	0.939	0.975	0.995	1.015	1.053					
Zygomycetes	23,130.0	0.968	0.993	1.004	1.015	1.032					
**Pollens**											
Malvids (*Acer, Tilia, Aesculus,* Cruciferae*)*^†^	23,126.8	0.933	0.956	0.968	0.980	1.003					
Lamiidis (*Fraxinus,* Oleaceae*, Plantago)*^†^	23,126.8	0.934	0.957	0.968	0.980	1.002					
Rosales (*Ulmus, Morus, Prunus,* Urticaceae*, Crataegus)*	23,128.1	0.944	0.966	0.977	0.989	1.010					
Grasses (Cyperaceae, Gramineae)	23,128.6	0.978	1.010	1.026	1.042	1.071					
Poales (Typhacaea)	23,128.9	0.932	0.960	0.975	0.989	1.015					
Proteales (*Platanus)*	23,128.9	0.944	0.971	0.984	0.996	1.016					
Malpighiales (*Salix, Populus*)	23,129.0	0.981	1.004	1.016	1.028	1.050					
Caryophyllales (Chenopodiaceae*, Rumex, Salsola pestifer)*	23,129.1	0.974	1.003	1.018	1.032	1.059					
Conifers (Cupressaceae*, Larix,* Pinaceae*, Tsuga)*	23,129.4	0.982	1.002	1.012	1.022	1.041					
Fagales (*Betula, Alnus, Corylus, Quercus, Fagus, Castanea)*	23,129.5	0.978	0.998	1.009	1.020	1.040					
Juglandaceae (*Juglans, Carya)*	23,130.0	0.962	0.985	0.996	1.007	1.028					
Campanulids (*Sambucus, Ambrosia, Solidago, Artemisia)*	23,130.1	0.955	0.986	1.003	1.020	1.028					
**Incidence of healthcare encounters for atopy (ICD codes)**											
Atopic dermatitis (L20)^†^	23,121.1	1.006	1.026	1.036	1.047	1.067	1.007	1.028	1.040	1.040	1.072
Asthma (J45)	23,127.7	0.951	0.982	0.998	1.015	1.047					
Rhinitis (J30)	23,128.5	0.934	0.959	0.972	0.986	1.011					
Urticaria (L50)	23,132.1	0.971	0.994	1.007	1.019	1.043					

**Incidence of healthcare encounters by infectious categories (ICD codes)**											
Viral intestinal infections (A08)^†^	23,120.0	1.017	1.041	1.054	1.066	1.091	1.028	1.054	1.068	1.082	1.108
Bacterial skin infections (A69.2, K05.0, L00–L03, L08)^†^	23,126.7	0.883	0.914	0.930	0.947	0.979	0.870	0.906	0.925	0.945	0.983
Viral LRTI (J09, J10, J11, J12, J17.1, J20.3/.4/.5/.6/.7/.8^^^, J21.0/.1/.8^^^)	23,127.5	0.955	0.976	0.987	0.997	1.017					
Viral URTI (B27, J00, J02.8^^^, J03.8^^^, J04^^^, J05.0^^^)	23,127.6	0.953	0.976	0.988	1.000	1.024					
Intestinal infections no organism identified (A06, A07, A09)	23,127.8	0.933	0.960	0.975	0.990	1.019					
Bacterial LRTI (A15, A16, J13, J14, J15, J16.0, J17.0, J20.0/.1/.2/.8^^^, J21.8^^^)	23,127.9	0.961	0.981	0.992	1.003	1.022					
Bacterial intestinal infections (A01–A05)	23,128.4	0.951	0.975	0.987	1.000	1.025					
Bacterial URTI (J02.0/.8^^^, J03.0/.8^^^, J04^^^, J05^^^, J36.0^^^)	23,129.4	0.888	0.918	0.933	0.949	0.980					
URTI no organism identified (J01, J02.8/.9, J03.8/.9, J04, J05.0, J06, J36, J39)	23,129.7	0.929	0.958	0.974	0.990	1.020					
Fungal infections (B37, J17.2)	23,129.9	0.900	0.930	0.946	0.962	0.993					
Viral skin infections (B00–B02, B05–B09)	23,129.9	0.909	0.940	0.957	0.974	1.007					
LRTI no organism identified (J16.8, J17.3/.8, J18, J20.8/.9, J21.8/.9, J22)	23,130.1	0.921	0.950	0.966	0.982	1.013					
**Incidence of healthcare encounters for specific pathogens (N of admissions)**											
*Mycoplasma* (8296)^†^	23,117.2	1.021	1.038	1.047	1.056	1.073	1.024	1.041	1.050	1.059	1.076
*Candida* (283,837)^†^	23,123.6	0.869	0.900	0.917	0.934	0.966	0.848	0.884	0.903	0.923	0.962
*Campylobacte*r (8368)^†^	23,123.6	0.996	1.015	1.025	1.036	1.055	1.005	1.025	1.036	1.047	1.067
Coronavirus (1658)^†^	23,124.2	1.000	1.014	1.020	1.027	1.040	0.998	1.011	1.018	1.025	1.038
Measles (2262)^†^	23,124.4	0.998	1.012	1.019	1.027	1.040	1.000	1.015	1.022	1.029	1.042
*Klebsiella* (120,905)^†^	23,124.8	0.983	1.011	1.027	1.042	1.073	1.003	1.036	1.053	1.071	1.105
*Haemophilus influenzae* (19,927)^†^	23,125.9	0.988	1.007	1.017	1.027	1.047					
*Salmonella* (16,585)^†^	23,126.0	0.991	1.009	1.019	1.029	1.048	0.990	1.009	1.019	1.029	1.048
*Clostridium* (214,593)^†^	23,126.1	0.920	0.947	0.962	0.976	1.005					
Herpes simplex (103,857)^†^	23,126.2	0.918	0.945	0.959	0.974	1.002					
*Borrelia* (6955)^†^	23,126.3	0.936	0.960	0.972	0.984	1.006					
Adenovirus (10,012)^†^	23,126.3	0.988	1.005	1.015	1.024	1.041					
Metapneumovirus (2655)^†^	23,126.6	0.957	0.975	0.983	0.992	1.008	0.952	0.970	0.979	0.987	1.004
Cytomegalovirus (8749)^†^	23,126.8	0.989	1.006	1.015	1.024	1.041					
*Pseudomonas* (103,288)†	23,127.0	0.930	0.957	0.971	0.986	1.014					
*Mycobacterium* (23,866)	23,127.0	0.959	0.979	0.990	1.001	1.021					
Molluscum contagiosum (10,336)	23,127.0	0.942	0.963	0.974	0.985	1.006					
Meningococcus (1839)	23,127.0	0.961	0.977	0.986	0.995	1.011					
Varicella (53,077)	23,127.0	0.972	0.995	1.007	1.019	1.042					
Papillomavirus (77,856)	23,127.3	0.922	0.951	0.966	0.981	1.008					
Mumps (10,498)	23,127.3	0.981	1.000	1.009	1.019	1.037					
*Bordetella* (13,630)	23,127.5	0.953	0.973	0.983	0.993	1.012					
Influenza (410,237)	23,127.6	0.957	0.976	0.986	0.996	1.014					
Epstein Barr Virus (6007)	23,127.7	0.974	0.990	0.999	1.007	1.024					
Herpes virus (5167)	23,127.7	0.977	0.994	1.003	1.012	1.029					
Parainfluenza (2490)	23,127.8	0.967	0.983	0.992	1.000	1.016					
Zoster (248,482)	23,127.8	0.891	0.921	0.937	0.953	0.985					
Hepatitis (2826)	23,127.8	0.981	0.997	1.005	1.013	1.028					
*Shigella* (1274)	23,127.8	0.984	0.998	1.006	1.014	1.028					
Norovirus (7507)	23,127.8	0.974	0.992	1.001	1.009	1.026					
Respiratory Syncytial Virus (86,764)	23,128.0	0.970	0.989	0.999	1.009	1.028					

Model/predictor (definition)	WAIC	Univariable SIR percentile					Multivariable SIR percentile				
		2.5th	25th	50th	75th	97.5th	2.5th	25th	50th	75th	97.5th
*Bacillus* (3224)	23,128.0	0.969	0.987	0.997	1.006	1.024					
*Proteus* (42,125)	23,128.0	0.958	0.981	0.993	1.005	1.028					
*Staphylococcus* (348,123)	23,128.0	0.941	0.973	0.991	1.008	1.043					
Rotavirus (11,846)	23,128.1	0.978	0.997	1.006	1.016	1.033					
Enterovirus (73,642)	23,128.2	0.955	0.977	0.989	1.001	1.024					
*Escherichia coli* (588,333)	23,128.4	0.915	0.947	0.965	0.983	1.017					
*Streptococcus* (348,123)	23,128.4	0.888	0.919	0.936	0.953	0.985					
Parvovirus (14,450)	23,128.6	0.952	0.973	0.984	0.995	1.016					

In the Bayesian model framework, a decrease in the Watanabe-Akaike information criterion (WAIC) is an indication of improved predictive accuracy. The simplest model in the table is the linear time model which consists of the spatially structured and unstructured term (Besag-York-Mollie model) and a linear time trend. A random walk of order 1 (temporally structured effect) and a seasonal cycle with length 12 months are sequentially added. The latter model shows the lowest WAIC and is therefore used as the base model. The WAICs for the adjustment factors (percentage of population age 0–4 and East Asians) were only listed for completeness, but not used as a selection criterion. In all models listed under ‘Main regression model’, candidate variables were added to the adjusted base model one-by-one and listed by ascending WAIC within the variable category. Variables which, when added to the adjusted base model, resulted in a model with a lower WAIC (> 1 change), were included in a preliminary multivariable model. These variables were further subject to selection through backward elimination based on them increasing the multivariable WAIC. The percentile landmarks of the posterior coefficients of the fixed effects for all univariable models (‘Screening’) and that were included in the multivariable model are presented with standardized incidence rate associated with a 1 standard deviation change in the predictor. The original units for each variable are indicated for general information.

*FSA* forward sortation area, *ICD* international classification of disease, *SIR* standardized incidence rate, *WAIC* Watanabe-Akaike Information Criterion.

^†^ Improved (i.e. lowered) WAIC by >1 over the adjusted based model.

^^^ICD code associated with a known organism indicate by ICD codes B95–B98.

### Weather and pollution data

Monthly weather data were obtained from the European Centre for Medium-Range Weather Forecasts (ECMWF) reanalysis ERA-Interim product. ERA-Interim uses an integrated forecasting system model and the 4D-variational data assimilation system^32^. We included variables that are known or suspected to directly impact human health, or influence disease vectors and/or the growth and transport of aeroallergens (see full list in Table 1). Air pollution data were obtained from the ECMWF Copernicus Atmospheric Monitoring Service (CAMS) Reanalysis [2018]^33^. The CAMS Reanalysis provides information on aerosol and reactive gases as total column values and are based on assimilation of satellite observations into a forecasting model. We included particulate matter smaller than 2.5 µm (PM2.5), nitrogen dioxide, ozone, sulfur dioxide and carbon monoxide from the CAMS reanalysis. Given their similar association with the incidence of KD, nitrogen dioxide and sulfur dioxide levels were combined into a single variable by summing up the two variables. The reanalysis data were obtained on a 1.25-degree longitude and 1.25-degree latitude grid, and values for grid cells with a center inside an FSA were aggregated to obtain data per FSA.

### Atmospheric biological particles

Daily measures of major biological atmospheric particles are collected by Aerobiology Research Laboratories (Ottawa, Canada) via rotational impaction sampling methods. These data are representative of the amount of environmental allergens in the atmosphere. Data were obtained for the 10 cities representing the largest populations in Canada (Vancouver, Edmonton, Calgary, Regina, Winnipeg, London, Toronto, Ottawa, Moncton and St. Johns). Previous studies have shown that biological atmospheric particle concentration can be regionalized up to ranges of ~ 100 km from the sampling site (~ 30,000 km^2^) for analysis^34^. As such, for each FSA, atmospheric biological particle data were assigned from the closest city (i.e. the closest particle collection point). These data are only available during the growing season of the year. Pollen counts before and after the growing season were set to zero.

### Data preparation for modelling

Due to the different scales and units of the covariates, all covariates were centered to mean zero and scaled to variance one before the analysis, except for wind direction which was not centered to conserve the wind direction.

### Aggregation of spatial units

All data except for weather and pollution data was originally available on an FSA level. FSA boundaries were obtained from the 2011 Canadian Census^35^. FSAs designate a geographical area based on the first 3 digits of the postal code (equivalent to the US zip code) that corresponds to an average of ~ 20,000 individuals. In order to maintain sufficient case density for analysis, Northern regions, where population density was extremely low, were excluded. The remaining FSAs were combined using a data driven strategy.

To begin with, the FSA with the smallest population (R1B with 10 inhabitants) was selected. FSAs that share at least one common boundary with this initial FSA were identified (R1A and R3C). The neighbouring FSA with the smallest population (R3C with 1620 population) was then combined to the initial FSA, i.e. the spatial polygons of R1B and R3C were aggregated by dissolving their internal boundaries. We obtain a new map, for which we have one new area (R1B ∩ R3C) with a population of 1630. From this new map, we again selected the FSA with the smallest population (V4G). This FSA had 5 neighbours from which the one with the smallest population was selected for spatial aggregation. This procedure was repeated until FSAs were combined into 100 groups. The supplementary map (online auxiliary material) shows the combined regions as well as lists of all FSAs that were aggregated for each region. Spatial aggregation was performed in order to preserve the granularity of the data and achieve sufficient case density for modelling.

### Bayesian disease model

A hierarchical Bayesian model framework is used to address the issues posed by the sparseness of the data and the spatial dependencies between the data^36,37^. The standardized incidence rate for KD in each area and month was modelled with the Besag-York-Mollie (BYM) model^38^, which includes spatial random effects that account for the spatial autocorrelation of the data. Temporal effects have been added to the model sequentially. We added a linear trend, dynamic non-linear trend^39^, specified through a random walk model of order 1, and a seasonal term. More details on the model parameterization can be found in the supplement.

To approximate posterior marginals, we used an integrated nested Laplace approximation (INLA) approach, which is a deterministic algorithm for Bayesian inference proposed by Rue et al.^40^ The computation was performed with the R-INLA interface^41^. To select the best temporal structure of the model and to select variables, we compared the Watanabe-Akaike information criterion (WAIC) of different models (see Table 1). The calculation of WAIC followed Gelman et al.^42^.

All statistical analyses and maps creation for this project were performed using R version 4.0.2 (https://www.R-project.org). Base map and data obtained from OpenStreetMap and OpenStreetMap Foundation (www.openstreetmap.org).

## Results

### KD incidence and spatial and temporal variation

**Figure 1:**
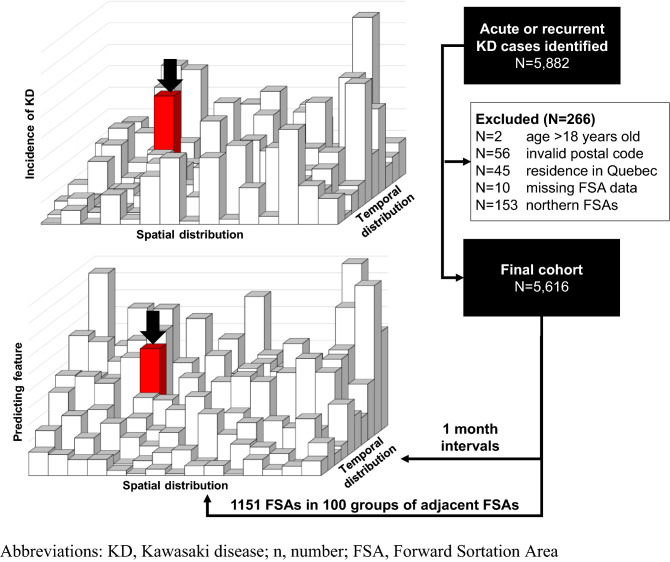
Identification of KD patients and theoretical framework of the spatiotemporal matrix for use for modelling. The spatial and temporal scales were held constant for incidence of KD and the distribution of all predictive features so that data points at the same position in the matrix are aligned with each other in time and space. The temporal (1 month) and spatial (group of ~ 10 FSAs) calipers were decided based on density distribution of the incidence of KD. The example below (red bar) shows the alignment of the incidence of KD and of a generic predicting feature at a specific time and location in our spatiotemporal matrix. *KD* Kawasaki disease, *n* number, *FSA* Forward Sortation Area.

From April 2004-March 2017, a total of 5,882 acute hospital admissions for KD were identified in the discharge abstract database maintained by the Canadian Institute of Health Information (CIHI) based on the ICD-10-CA standard code M30.3, of which 266 were excluded according to our previously designed algorithm to identify readmissions and between-hospital transfers (Fig. 1)^22^. The final number of acute admissions included in this study was 5,616, with an annual incidence of 22.3 per 100,000 children under the age of four, 7.2 per 100,000 children from 5 to 9 years of age and 0.8 per 100,000 children from 10 to 18 years of age. Spatiotemporal variations of KD were modelled with a Bayesian hierarchical model (see *Expanded methods* and *Supplementary information* for details), which includes covariates related to environmental exposure (list in Table 1). We compared several spatiotemporal models that accounted for the temporal structure differently, but were all based on the Besag-York-Mollie model to account for spatial autocorrelation^38^. The final multivariable model included a linear and non-linear time component, and covariates that improved the performance of the model.

**Figure 2:**
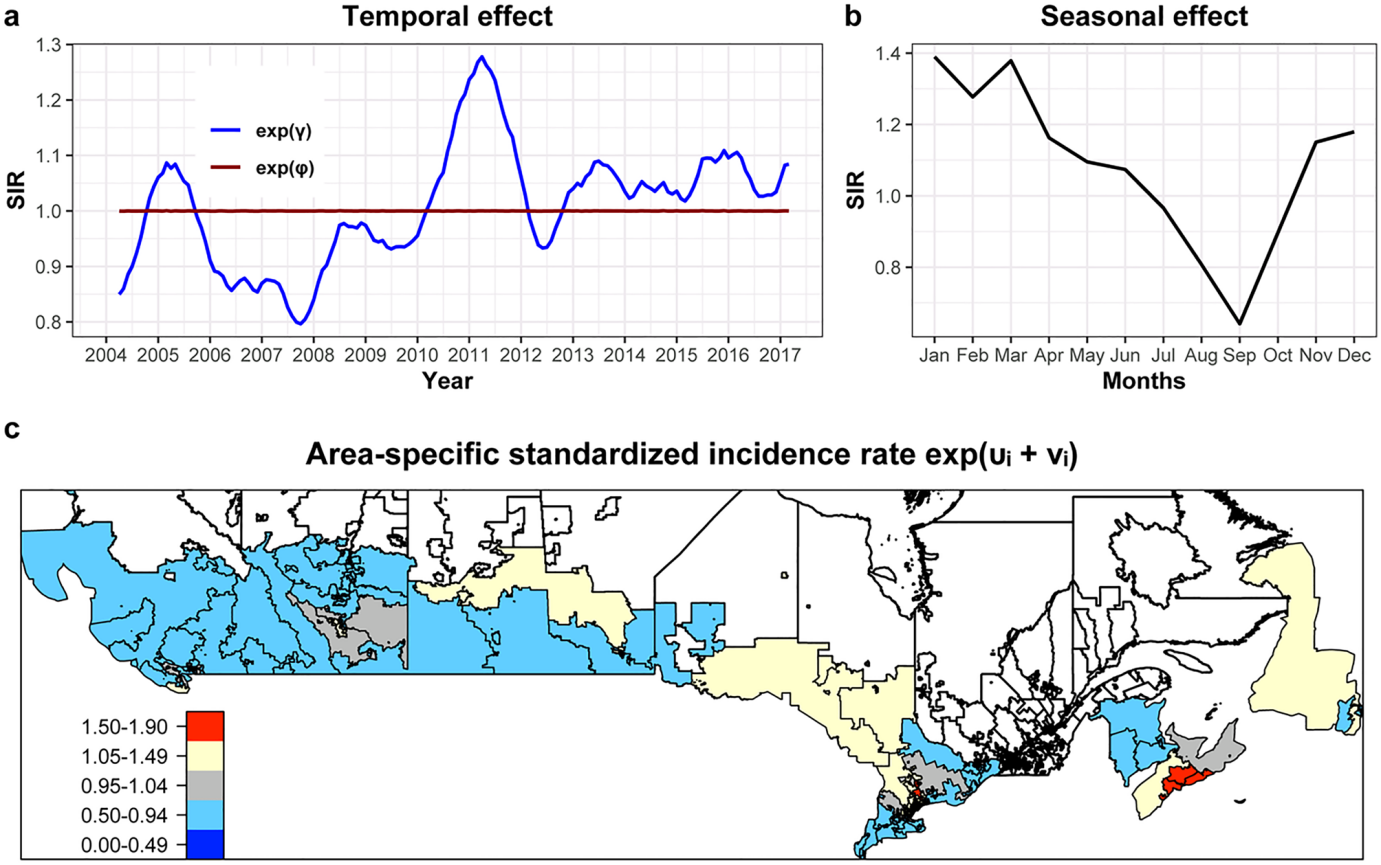
Posterior mean value for the temporal and spatial effects for the risk of Kawasaki disease (KD) in Canada from April 2004 to March 2017 based on the Bayesian hierarchical model. Standardized incidence rate above 1 indicate increased risks, and below 1 decreased risks. (**a**) Monthly temporal unstructured and structured trends. An increasing trend is visible for the unstructured effect (solid line) across time. The structured effect (dashed line) shows nearly no fluctuation. (**b**) The seasonal effect illustrates a below average standardized incidence rate for KD in September and a high risk in the winter months. (**c**) Mean spatially autocorrelated random effect, expressed as area-specific standardized incidence rate. Areas with standardized incidence rate larger than 1 have an above average risk compared to the rest of Canada, while standardized incidence rate smaller than 1 indicate a below average risk. The red colours in the Greater Toronto Area and around Halifax for example indicate a 50 to 90% increase of risk. For better visibility of small regions, an interactive map can be found in the online appendix. The spatial units (of the analysis regions consisting of several forward sorting areas) are indicated with boundaries and grey filling. White regions were added to the plot for easier orientation. For uncertainty estimates, see online appendix.

Standardized incidence rates obtained from the mean of the posterior distribution of the temporal and spatial random effects are presented in Fig. 2. The model suggests that the risk for KD was highest between late 2010 and 2011, and reached its lowest point at the end of 2007 (Fig. 2A). As expected, a distinct seasonal distribution with higher risk for KD during the winter months and relatively lower risks at the end of the summer months was found (Fig. 2B). Spatial areas with increased risk for KD were identified in some areas around Toronto and Halifax (spatial standardized incidence rate of larger 1.5 and 1.8 respectively), with a posterior probability > 99% (Fig. 2C). Further hotspots were found in region of Northern Ontario and the city of Calgary (spatial standardized incidence rate > 1.3). A detailed map including posterior probabilities is provided in Appendix 2.

### Environmental factors and exposures associated with distribution of KD

**Figure 3:**
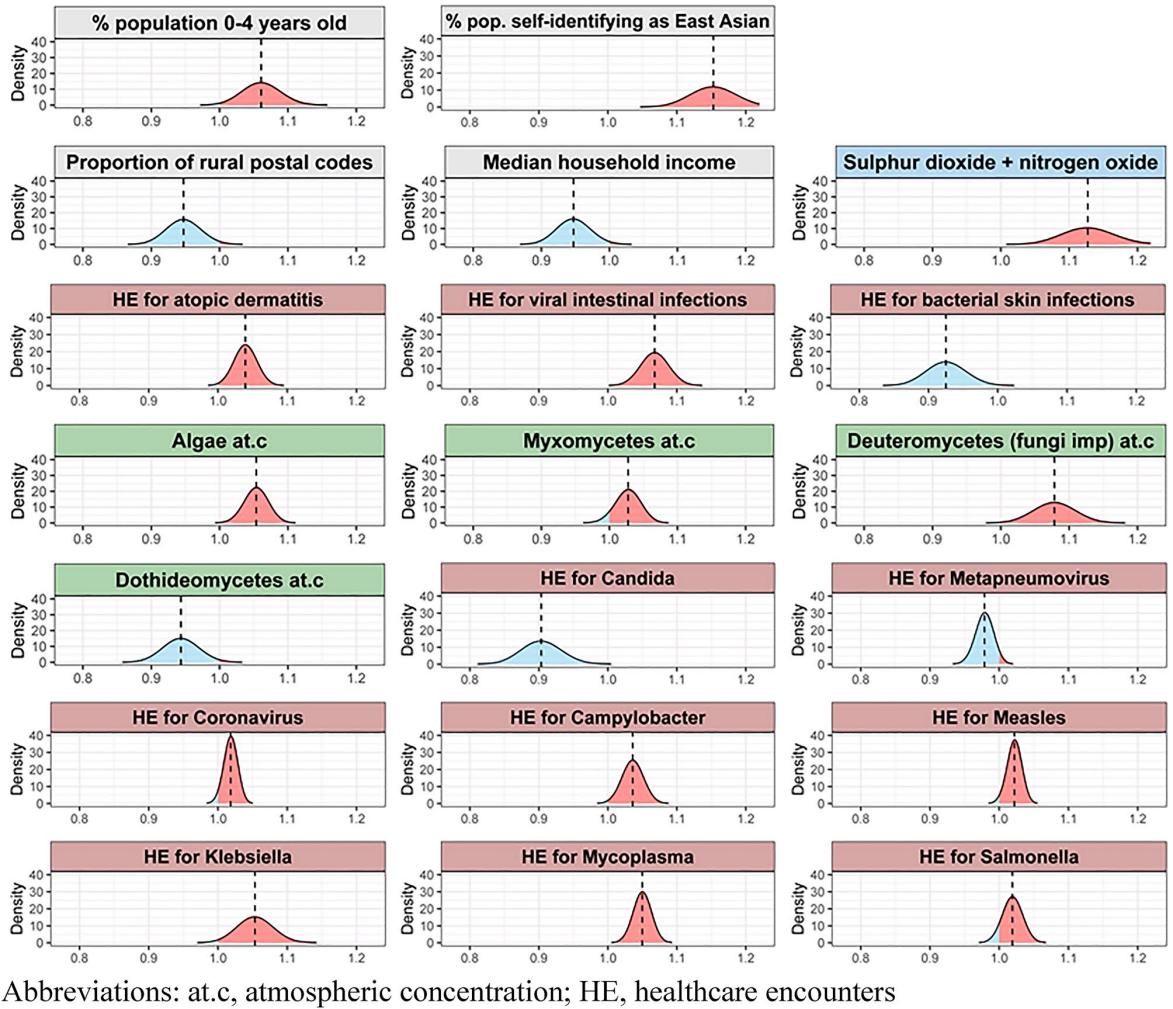
Association of factors with the standardized incidence rate for Kawasaki disease (KD). Distribution of the standardized incidence rate (line = median) based on the posteriors of the fixed effects (same as multivariable results in Table 1). The values represent the change in standardized incidence rates for a 1 standard deviation change in the predictor. A value below 1.0 (blue) indicates a negative association between the predictor and the standardized incidence rate for KD, and values above 1.0 (red) indicate a positive association. The background color of the labels indicates the type of predictor, with grey representing genetic and demographic factors, blue representing atmospheric pollution, red representing the incidence of healthcare encounters for various causes and green representing atmospheric concentration of biological particles. *at.c* atmospheric concentration, *HE* healthcare encounters.

For each of the covariates, a base model that included only spatial and temporal effects and adjusted for distribution of age and ethnicity in the population was compared with models that additionally included one of the covariates of interest based on model fit, measured by the Watanabe-Akaike information criteria (WAIC). The WAIC for the base model was 23,134.0, while the adjusted base model WAIC was 23,128.0 (see top of Table 1). Only variables that reduced the WAIC from the base model by more than 1, i.e. improved the model fit, were included in a multivariable model. In a second step, the deletion of each variable from this multivariable model was tested in a backward selection to identify and remove variables that did not contribute to the multivariable model fit (see Table 1 and Fig. 3).

Both adjustment factors, the percentage of population under 4 years of age and the percentage of population self-identifying as Taiwanese, Korean, Japanese or Chinese (‘East Asian’) were associated with increased risk for KD. In the final multivariable model, environmental factors contributing to an increased risk of KD included lower median income, lower proportion of an area classified as rural, increased atmospheric concentration of spores from Myxomycetes (slime molds), Deuteromycetes (fungi imperfecti) and algae concentrations, lower atmospheric concentration of Dothideomycetes spores and higher combined smog (NO_2_ + SO_2_) concentration in the atmosphere. Increased incidence of KD was also associated with higher incidence of healthcare encounters for atopic dermatitis, viral intestinal illnesses, bacterial pneumonia (associated with *Mycoplasma*, *Klebsiella* and possibly *Haemophilus influenzae*), and multisystem infections with a heterogeneous group of pathogens known to be potentially associated with hyperinflammatory or autoimmune syndromes (*Campylobacter*, cytomegalovirus, coronavirus, measles, *Salmonella*). Higher incidence of healthcare encounters for bacterial skin infections or fungal infection secondary to *Candida* were associated with reduced incidence of KD. The WAIC for the final multivariable model was 23,059.7.

### Correlation between modelled and observed KD rates

**Figure 4:**
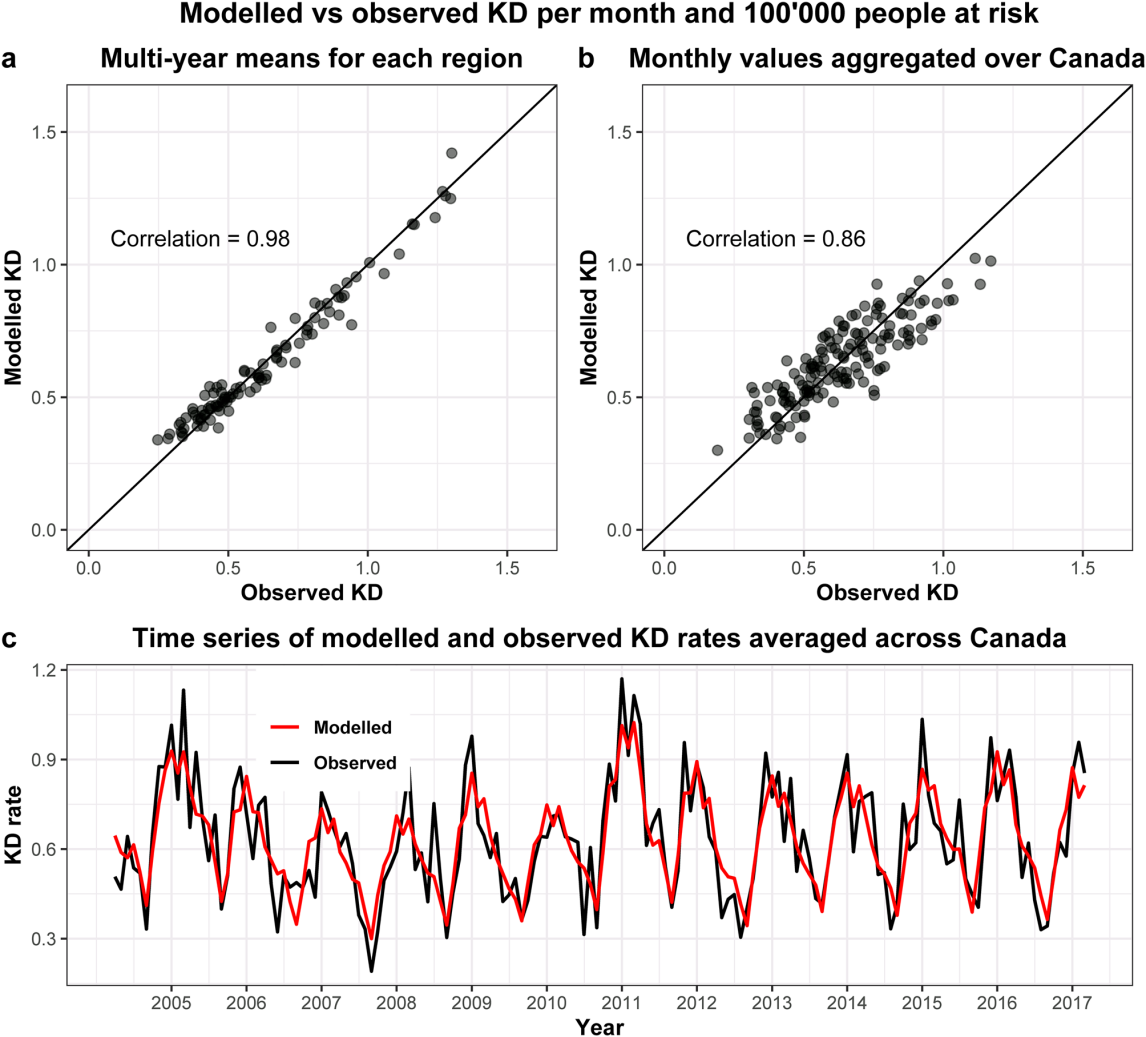
Correlation between observed and modelled Kawasaki disease (KD) rates. Modelled rates have been obtained from the exponentiated risk ratios of the multivariable hierarchical Bayesian model posteriors, multiplied by the expected rate. (**a**) Multi-year mean of observed and modelled KD rates, where each dot represents 1 of the 100 regions (groups of forward sorting areas). (**b**) Regional mean of observed and modelled KD rates. The rates were aggregated across all of Canada, and each dot represents 1 month of the 13 years. (**c**) Time series of observed (black) vs modelled (red) KD rates averaged over Canada from April 2004 to March 2017.

The correlation between the multi-year mean of observed and modelled KD incidence across the regions was 0.98 (Fig. 4A). The correlation across time was 0.86 (Fig. 4B). The time series of the observed and modelled KD incidences averaged across Canada (Fig. 4C) shows that the model reproduced the temporal variation well. Adding environmental factors, aeroallergen exposure and infectious agents to the model improved the observed-to-modelled correlation across years and provinces compared to our model that only adjusted to the population numbers, age and ethnicity distribution (correlation of 0.56 vs. 0.47).

## Discussion

**Figure 5:**
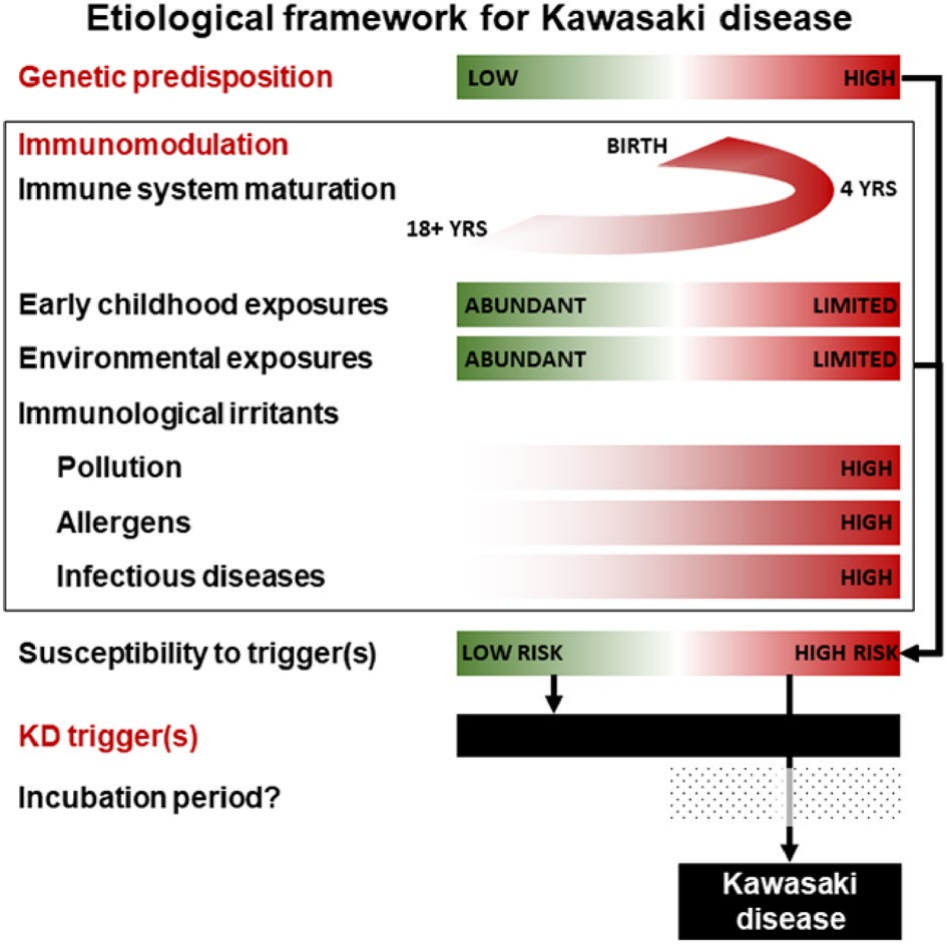
Hypothetical framework of Kawasaki disease (KD) etiology and risk based on published evidence, our previously published environmental case control study^21^ and the evidence presented in our current spatiotemporal model of the spatiotemporal distribution of KD. Red indicates higher risk while green indicates lower risk.

In this study, a mathematical model of the spatiotemporal distribution of KD in Canada over a 13-year period was derived. Factors found to be associated with the spatiotemporal incidence of KD included numerous environmental and infectious factors which were integrated into a comprehensive model for the distribution of KD. This study is significant both from the mathematical approach used to combine numerous dimensions of risk measured on different scales into a single model and by the high degree with which it could recapitulate the spatiotemporal distribution of KD. Such a model could eventually form the basis of a prediction and surveillance model for the future incidence of KD in a specific location. Careful examination of associated factors and how they contribute to the risk of KD can also substantially contribute to our understanding of the etiology and pathogenesis of KD (Fig. 5).

Some well known features of the epidemiology of KD were readily apparent in the associations observed in this study, including an increased risk in children 0–4 years old (although up to 30% of cases occur after 5 years of age), presence of seasonal patterns^14^ and spatiotemporal clusters^17–19^, as well as a higher risk in people of East Asian ancestry^11^. Numerous additional environmental factors were also identified in this study, some of which had already been reported in previous studies. Without a known etiological agent, epidemiological patterns and associations can be used to extrapolate some aspects of the etiology and pathogenesis of the disease. Positing a framework where KD is an immunological reaction to a still unidentified trigger(s) in genetically and/or immunologically susceptible individuals, epidemiological patterns and associations can reflect the inherent susceptibility of the population, the local or global distribution of the trigger or can be reflecting the presence of factor(s) modulating the risk of developing KD when encountering said trigger(s). Some environmental factors identified in this study might fit those scenarios and are discussed below.

Beyond the genetic susceptibility to KD, which is evidenced by the strong association between Asian ancestry and various genetic polymorphisms and increased risk of KD^11^, other factors might increase the inherent susceptibility of a population to KD through early childhood experience. It has been proposed that KD partially develops through the hygiene hypothesis, where lack of early microbial experience and limited exposure to allergens may promote atopy and predisposes children to a hyper-reactive response to a normal trigger^43^. A study in Japan reported that children living in households with up to three persons had a 1.6 fold increased risk of developing KD compared to those from households with more than five persons^16^.

The seasonal pattern of KD noted in this study was concordant with previous findings^22,44^. Seasonal variations in KD have been the subject of multiple previous studies, and is one of the most consistently described epidemiological features^14^. Multiple environmental factors found to be associated with the incidence of KD in this study also have a seasonal distribution. Thus, these may contribute, in a multifactorial manner, to the seasonal distribution of KD.

Results from previous studies have shown that some environmental exposures might be associated with the risk of KD, either positively or negatively. Several studies have reported higher incidences of KD associated with urbanized settings which might reflect the presence of modulating factors^16,21,45^. In a previous case–control epidemiological study we showed that children living in areas with more trees, farms and closer proximity to a natural body of water were at lower risk of KD than children living in areas with reduced exposures^21^. In this study, some of the factors identified for KD are consistent with these previous findings, including the presence of spatial hotspots in cities, decreased risk in rural areas and in environment with denser vegetation and increased atmospheric concentration of some biological particles.

At the same time, activation of the inflammasome via various environmental exposures might be increasing the risk of KD^46^. One such example is pollution exposure. In this study, an association between higher atmospheric concentration of SO_2_ and NO_2_ and increased risk of KD was noted. A recent Japanese publication reported that a 1-μg/m^3^ increase in NO and SO_2_ were associated with a higher incidence of KD (3.94, 95% CI 0.04–7.98 and 3.60, 95% CI 1.12–6.14 respectively)^47^. Air pollution has also been shown to negatively affect the respiratory mucosa through an interaction with several defense mechanisms leading to increased mucous production, epithelial barrier dysfunction and attenuated cytokine response^48–50^. The association between lower household income and increasing KD risk found in this study could also be related to activation of the inflammasome. Children from lower socioeconomic areas are known to have generally greater exposure to pollution, dust, toxins and allergens, and are at higher risk of multiple atopic and inflammatory disease, such as asthma and KD because of those exposures^16,51,52^.

Beyond susceptibility, case distribution and modulation, environmental associations might point to potential targets for the trigger(s). Higher atmospheric levels of Deuteromycetes (fungi imperfecti) and Myxomycetes (slime molds), both spore-producing, were found to be associated with higher incidences of KD, as were greater atmospheric concentrations of algal spores. Many spore-producing fungi and some algae have allergenic properties, and are known to be capable of initiating an immune response in human in addition to activating the inflammasome^53,54^. In a mouse model mimicking KD, intraperitoneal injection of *Candida albicans* extract has been shown to induce coronary arteritis^55^. Fungi are also capable of producing MAMPs such as beta-glucans and chitin, which may stimulate an inappropriate immune response through pattern recognition receptors^56^.

There is also a longstanding recognition that a substantial number of patients with KD present either with a documented infection, a recent history of an infectious illness or with infectious disease symptoms^57^. Numerous previous studies have postulated that one or more infectious agents is/are the trigger for KD, but no consistent or definitive findings have been reported^58^. Both respiratory and gastrointestinal diseases have been reported with KD and, indeed, in a previous case–control study, children with KD were found to exhibit respiratory and gastrointestinal symptoms in a higher proportion than controls up to 8–31 days before the appearance of the first KD symptoms^21^.

In this study, we identified groups of healthcare encounters for infections that were associated with increased incidence of KD. One interesting finding is the association between viral intestinal infections and increased incidence of KD. In North America, the most common causes of viral gastroenteritis (i.e. rotavirus, adenovirus and norovirus infections) exhibit a winter peak; notably in our previous environmental case–control study, rotavirus vaccination was associated with a lower risk of KD^21^. However, an autumn or spring peak is seen in other parts of the world, consistent with the global epidemiology of KD^59^. The association between viral intestinal infections and increased incidence of KD possibly reflect the facilitation of the entry of the trigger into the blood stream and as such would represent a modulating factor as opposed to a potential trigger. This being said, in the absence of direct experimentation, it remains unknown whether spores or infections can actually trigger KD or whether they are additional modulators.

This study must be viewed in light of some limitations. Firstly, this is an observational epidemiological study and, as such, associations are ecologic in nature. Any causal inferences are implied. Secondly, some dimensions of environmental risk dimensions which have previously been linked to KD, such as the microbiome, could not be included in our population-level model. Third, we were limited regarding the granularity of our model, given that KD is a rare disease and cases are dispersed in space and time. Therefore, any smaller, unpredicted changes in factor levels displayed over days or hours would not be captured. In addition, Census data were only available every 5 years, as such we were not able to fully adjust for the temporal variation in ethnicity across Canada across our study period, but fluctuations were expected to be minor. All spatial effects were regionalized, which meant that local environmental factors could not be considered. Moreover, atmospheric biological data were only available for 10 stations situated across Canada, but were extrapolated to the nearest FSA region for each of the 100 FSA groups. This limitation may potentially impact the validity of our pollen findings, as pollen dispersal is likely a local phenomenon. While KD incidence varies strongly with age, we cannot exclude that some factors may be more relevant to certain age groups than others, although no evidence of such an effect has been previously reported. The lower number of patients with KD > 5 years of age precluded a sub-analysis stratified by age. Finally, it is important to note that our proposed theoretical framework is a hypothesis and should not be construed as being confirmed.

## Conclusion

Our model of the spatiotemporal distribution of KD across Canada over a 13-year period was able to explain a substantial portion of the variation in the incidence of KD over that time period and identified multiple environmental factors that could be integrated into a theoretical etiological and pathophysiological framework for the spatiotemporal distribution of the incidence of KD. This framework includes a child’s susceptibility to the disease, the presence of modulating factors and the spatiotemporal distribution of the trigger(s) and/or of modulating factors. Findings from this study strengthen our understanding of the epidemiology of KD, which is likely affected by various factors reflecting the complex reality of KD.

## Supplementary Information

Supplementary Information 1.

Supplementary Information 2.

## Data Availability

Both the Hospital for Sick Children Ethics Committee and the Canadian Institute for Health Information have placed legal restrictions on sharing the data used in this study. The data from this study contain personal health information and as such, disclosure and distribution, even in an anonymized format, is restricted under the Ontario Personal Health Information Protection Act (PHIPA). Some data will only be available until March 31, 2025 after which it must be destroyed as per the Non-Disclosure/Confidentiality Agreement required by the Canadian Institute for Health Information. Data used in this study can be accessed by qualified researchers who meet the criteria for access to confidential health information. In addition to contacting the PI to access the data, requestors will be required to obtain approval from the Hospital for Sick Children Ethics Committee and the Canadian Institute for Health Information Data Access Program. The authors are legally prohibited from disclosing the raw aeroallergen data; interested parties would need to obtain this data directly from Aerobiology Research Laboratories (Ottawa, Canada).

## References

[1] McCrindle BW, Rowley AH, Newburger JW, Burns JC, Bolger AF, Gewitz M, Baker AL, Jackson MA, Takahashi M, Shah PB, Kobayashi T, Wu MH, Saji TT, Pahl E (2017). Diagnosis, treatment, and long-term management of kawasaki disease: A scientific statement for health professionals From the American Heart Association. Circulation.

[2] McCrindle BW, Manlhiot C, Newburger JW, Harahsheh AS, Giglia TM, Dallaire F, Friedman K, Low T, Runeckles K, Mathew M, Kutty S, Yetman AT, Raghuveer G, Pahl E, Norozi K, McHugh KE, Li JS, De Ferranti SD, Dahdah N (2020). Medium-term complications associated with coronary artery aneurysms after Kawasaki Disease: A study from the International Kawasaki Disease Registry. J Am Heart Assoc.

[3] Singh S, Vignesh P, Burgner D (2015). The epidemiology of Kawasaki disease: a global update. Arch. Dis. Child..

[4] Uehara R, Belay ED (2012). Epidemiology of Kawasaki disease in Asia, Europe, and the United States. J. Epidemiol..

[5] Chahal N, Somji Z, Manlhiot C, Clarizia NA, Ashley J, Yeung RS, McCrindle BW (2012). Rate, associated factors and outcomes of recurrence of Kawasaki disease in Ontario, Canada. Pediatr. Int..

[6] Onouchi Y (2009). Molecular genetics of Kawasaki disease. Pediatr. Res..

[7] Weng KP, Ho TY, Chiao YH, Cheng JT, Hsieh KS, Huang SH, Ou SF, Liu KH, Hsu CJ, Lu PJ, Hsiao M, Ger LP (2010). Cytokine genetic polymorphisms and susceptibility to Kawasaki disease in Taiwanese children. Circ. J..

[8] Onouchi Y, Gunji T, Burns JC, Shimizu C, Newburger JW, Yashiro M, Nakamura Y, Yanagawa H, Wakui K, Fukushima Y, Kishi F, Hamamoto K, Terai M, Sato Y, Ouchi K, Saji T, Nariai A, Kaburagi Y, Yoshikawa T, Suzuki K, Tanaka T, Nagai T, Cho H, Fujino A, Sekine A, Nakamichi R, Tsunoda T, Kawasaki T, Nakamura Y, Hata A (2008). ITPKC functional polymorphism associated with Kawasaki disease susceptibility and formation of coronary artery aneurysms. Nat. Genet..

[9] Uehara R, Yashiro M, Nakamura Y, Yanagawa H (2003). Kawasaki disease in parents and children. Acta Paediatr..

[10] Fujita Y, Nakamura Y, Sakata K, Hara N, Kobayashi M, Nagai M, Yanagawa H, Kawasaki T (1989). Kawasaki disease in families. Pediatrics.

[11] Onouchi Y (2012). Genetics of Kawasaki disease: what we know and don't know. Circ. J..

[12] Khor CC, Davila S, Shimizu C, Sheng S, Matsubara T, Suzuki Y, Newburger JW, Baker A, Burgner D, Breunis W, Kuijpers T, Wright VJ, Levin M, Hibberd ML, Burns JC (2011). Genome-wide linkage and association mapping identify susceptibility alleles in ABCC4 for Kawasaki disease. J. Med. Genet..

[13] Awaya A, Sahashi N (2004). The etiology of Kawasaki disease: does intense release of pollen induce pollinosis in constitutionally allergic adults, while constitutionally allergic infants develop Kawasaki disease?. Biomed. Pharmacother..

[14] Burns JC, Herzog L, Fabri O, Tremoulet AH, Rodo X, Uehara R, Burgner D, Bainto E, Pierce D, Tyree M, Cayan D (2013). Seasonality of Kawasaki disease: A global perspective. PLoS ONE.

[15] Nagao Y, Urabe C, Nakamura H, Hatano N (2016). Predicting the characteristics of the aetiological agent for Kawasaki disease from other paediatric infectious diseases in Japan. Epidemiol. Infect..

[16] Fujiwara T, Shobugawa Y, Matsumoto K, Kawachi I (2019). Association of early social environment with the onset of pediatric Kawasaki disease. Ann. Epidemiol..

[17] Burns JC, DeHaan LL, Shimizu C, Bainto EV, Tremoulet AH, Cayan DR, Burney JA (2021). Temporal clusters of Kawasaki disease cases share distinct phenotypes that suggest response to diverse triggers. J. Pediatr..

[18] Rypdal M, Rypdal V, Burney JA, Cayan D, Bainto E, Skochko S, Tremoulet AH, Creamean J, Shimizu C, Kim J, Burns JC (2018). Clustering and climate associations of Kawasaki Disease in San Diego County suggest environmental triggers. Sci. Rep..

[19] Hearn J, McCrindle BW, Mueller B, O'Shea S, Bernknopf B, Labelle M, Manlhiot C (2018). Spatiotemporal clustering of cases of Kawasaki disease and associated coronary artery aneurysms in Canada. Sci. Rep..

[20] Lee KY, Han JW, Lee JS (2007). Kawasaki disease may be a hyperimmune reaction of genetically susceptible children to variants of normal environmental flora. Med. Hypotheses.

[21] Manlhiot C, Mueller B, O’Shea S, Majeed H, Bernknopf B, Labelle M, Westcott KV, Bai H, Chahal N, Birken CS, Yeung RSM, McCrindle BW (2018). Environmental epidemiology of Kawasaki disease: Linking disease etiology, pathogenesis and global distribution. PLoS ONE.

[22] Manlhiot C, O'Shea S, Bernknopf B, LaBelle M, Chahal N, Dillenburg RF, Lai LS, Bock D, Lew B, Masood S, Mathew M, McCrindle BW (2018). Epidemiology of Kawasaki Disease in Canada 2004 to 2014: Comparison of surveillance using administrative data vs periodic medical record review. Can. J. Cardiol..

[23] Statistics Canada. *Census Forward Sortation Area Boundary File, Reference Guide*. (Canada, 2017).

[24] Statistics Canada. *2006 Census Profile of Age and Sex for Canada, Provinces, Territories and Forward Sortation Areas*. Catalogue No: 94-575-XCB2006003. (Canada, 2006).

[25] Statistics Canada. *Profile of Ethnic Origin and Visible Minorities for Canada, Provinces, Territories and Forward Sortation Areas*. Catalogue No: 94-580-XCB2006003. (Canada, 2006).

[26] Statistics Canada. *2006 Census Profile for Canada, Provinces, Territories and Forward Sortation Areas*. Catalogue No: 94-581-XCB2006003. (Canada, 2006).

[27] Statistics Canada. *Age (131) and Sex (3) for the Population of Canada and Forward Sortation Areas, 2011 Census*. Catalogue No: 98-311-XCB2011022. (Canada, 2011).

[28] Statistics Canada. *Age (in Single Years) and Average Age (127) and Sex (3) for the Population of Canada and Forward Sortation Areas, 2016 Census: 100% Data*. Catalogue No: 98-400-X2016008. (Canada, 2016).

[29] Statistics Canada. *2011 National Household Survey Profile*. Catalogue No: 99–004-X. (Canada, 2011).

[30] Statistics Canada. *Cartographic Boundary Files (CBF), 2006 Census*. Derived from the FSA cartographic boundary file by G. Romme. (Canada, 2006).

[31] Statistics Canada. *Ethnic Origin Reference Guide*. Vol. 1 (Canada, 2017).

[32] Dee DP, Uppala SM, Simmons AJ, Berrisford P, Poli P, Kobayashi S, Andrae U, Balmaseda MA, Balsamo G, Bauer P, Bechtold P, Beljaars ACM, van de Berg L, Bidlot J, Bormann N, Delsol C, Dragani R, Fuentes M, Geer AJ, Haimberger L, Healy SB, Hersbach H, Holm EV, Isaksen L, Kallberg P, Kohler M, Matricardi M, McNally AP, Monge-Sanz BM, Morcrette JJ, Park BK, Peubey C, de Rosnay P, Tavolato C, Thepaut JN, Vitart F (2011). The ERA-Interim reanalysis: Configuration and performance of the data assimilation system. Q. J. R. Meteorol. Soc..

[33] Inness A, Ades M, Agustí-Panareda A, Barré J, Benedictow A, Blechschmidt A-M, Dominguez JJ, Engelen R, Eskes H, Flemming J, Huijnen V, Jones L, Kipling Z, Massart S, Parrington M, Peuch V-H, Razinger M, Remy S, Schulz M, Suttie M (2019). The CAMS reanalysis of atmospheric composition. Atmos. Chem. Phys..

[34] Darrow LA, Hess J, Rogers CA, Tolbert PE, Klein M, Sarnat SE (2012). Ambient pollen concentrations and emergency department visits for asthma and wheeze. J. Allergy Clin. Immunol..

[35] Statistics Canada. *Census Forward Sortation Area Boundary File, Reference Guide*. (Canada, 2013).

[36] Best N, Richardson S, Thomson A (2005). A comparison of Bayesian spatial models for disease mapping. Stat. Methods Med. Res..

[37] Lawson AB (2014). Hierarchical modeling in spatial epidemiology. Wires Comput. Stat.

[38] Besag J, York J, Mollie A (1991). Bayesian image-restoration, with 2 applications in spatial statistics. Ann. Stat. Math..

[39] Knorr-Held L (2000). Bayesian modelling of inseparable space-time variation in disease risk. Stat. Med..

[40] Rue H, Martino S, Chopin N (2009). Approximate Bayesian inference for latent Gaussian models by using integrated nested Laplace approximations. J. R. Stat. Soc. B.

[41] Blangiardo, M. & Cameletti, M. Spatial and Spatio-temporal Bayesian Models with R-INLA Introduction. *Spatial and Spatio-Temporal Bayesian Models with R-Inla*, 1–18 (2015). 10.1016/j.sste.2016.03.001 27494955

[42] Gelman A, Hwang J, Vehtari A (2014). Understanding predictive information criteria for Bayesian models. Stat. Comput..

[43] Prokopakis E, Vardouniotis A, Kawauchi H, Scadding G, Georgalas C, Hellings P, Velegrakis G, Kalogjera L (2013). The pathophysiology of the hygiene hypothesis. Int. J. Pediatr. Otorhinolaryngol..

[44] Lin YT, Manlhiot C, Ching JC, Han RK, Nield LE, Dillenburg R, Pepelassis D, Lai LS, Smythe JF, Chahal N, Yeung RS, McCrindle BW (2010). Repeated systematic surveillance of Kawasaki disease in Ontario from 1995 to 2006. Pediatr. Int..

[45] Prakash J, Singh S, Gupta A, Bharti B, Bhalla AK (2016). Sociodemographic profile of children with Kawasaki disease in North India. Clin. Rheumatol..

[46] Bauer RN, Diaz-Sanchez D, Jaspers I (2012). Effects of air pollutants on innate immunity: the role of Toll-like receptors and nucleotide-binding oligomerization domain-like receptors. J. Allergy Clin. Immunol..

[47] Fujii F, Egami N, Inoue M, Koga H (2020). Weather condition, air pollutants, and epidemics as factors that potentially influence the development of Kawasaki disease. Sci. Total Environ.

[48] Mookherjee N, Piyadasa H, Ryu MH, Rider CF, Ezzati P, Spicer V, Carlsten C (2018). Inhaled diesel exhaust alters the allergen-induced bronchial secretome in humans. Eur. Respir. J..

[49] Zarcone MC, van Schadewijk A, Duistermaat E, Hiemstra PS, Kooter IM (2017). Diesel exhaust alters the response of cultured primary bronchial epithelial cells from patients with chronic obstructive pulmonary disease (COPD) to non-typeable Haemophilus influenzae. Respir. Res..

[50] Alphonse MP, Duong TT, Shumitzu C, Hoang TL, McCrindle BW, Franco A, Schurmans S, Philpott DJ, Hibberd ML, Burns J, Kuijpers TW, Yeung RS (2016). Inositol-triphosphate 3-kinase C mediates inflammasome activation and treatment response in Kawasaki disease. J. Immunol..

[51] Thakur N, Oh SS, Nguyen EA, Martin M, Roth LA, Galanter J, Gignoux CR, Eng C, Davis A, Meade K, LeNoir MA, Avila PC, Farber HJ, Serebrisky D, Brigino-Buenaventura E, Rodriguez-Cintron W, Kumar R, Williams LK, Bibbins-Domingo K, Thyne S, Sen S, Rodriguez-Santana JR, Borrell LN, Burchard EG (2013). Socioeconomic status and childhood asthma in urban minority youths: The GALA II and SAGE II studies. Am. J. Respir. Crit. Care Med..

[52] Harnden A, Mayon-White R, Perera R, Yeates D, Goldacre M, Burgner D (2009). Kawasaki disease in England: Ethnicity, deprivation, and respiratory pathogens. Pediatr. Infect. Dis. J..

[53] Levetin E, Horner WE, Scott JA, Workgrp EA (2016). Taxonomy of allergenic fungi. J. Aller. Cl. Immun. Pract..

[54] Tesson SVM, Skjoth CA, Santl-Temkiv T, Londahl J (2016). Airborne microalgae: Insights, opportunities, and challenges. Appl. Environ. Microbiol..

[55] Murata H (1979). Experimental candida-induced arteritis in mice: Relation to arteritis in the mucocutaneous lymph node syndrome. Microbiol. Immunol..

[56] Lewis LE, Bain JM, Lowes C, Gillespie C, Rudkin FM, Gow NA, Erwig LP (2012). Stage specific assessment of *Candida albicans* phagocytosis by macrophages identifies cell wall composition and morphogenesis as key determinants. PLoS Pathog..

[57] Benseler SM, McCrindle BW, Silverman ED, Tyrrell PN, Wong J, Yeung RS (2005). Infections and Kawasaki disease: Implications for coronary artery outcome. Pediatrics.

[58] Principi N, Rigante D, Esposito S (2013). The role of infection in Kawasaki syndrome. J. Infect..

[59] Cook SM, Glass RI, LeBaron CW, Ho MS (1990). Global seasonality of rotavirus infections. Bull. World Health Organ..

