# Supplementary material for: Associations between the spatiotemporal distribution of Kawasaki disease and environmental factors: evidence supporting a multifactorial etiologic model
Source: Sci Rep. 2021 Jul 16;11:14617. doi: 10.1038/s41598-021-93089-9 (PMC8285427; doi:10.1038/s41598-021-93089-9)
Supplement: Supplementary file 1 — Supplementary Information 1. [file 41598_2021_93089_MOESM1_ESM.docx]

**Supplementary information**

**Associations between the Spatiotemporal Distribution of Kawasaki Disease and Environmental Factors: Evidence Supporting a Multifactorial Etiologic Model**

**Low et al.**

**Supplementary Figure 1:** Study area, spatial aggregation and spatial risk. Each of the 100 study regions and their area-specific standardized incidence rate for Kawasaki Disease (KD), based on the posterior from the multivariable model, are shown in color. For each region, a table can be seen (by clicking on the region) with the following information:

1. Aggregated FSAs: The forward sortation areas (FSAs) that were aggregated into the region for analysis.
2. Area-specific standardized incidence rates (SIR): Area-specific standardized incidence rate, based on the mean of the posterior distribution of the spatial random effect ($\zeta_{i}= exp(u_{i}+\upsilon_{i}$), with $\upsilon_{i}$ being the spatially unstructured and $u_{i}$ the structured component). A SIR larger than 1 indicates that the region has a higher risk compared to the rest of the study area, while a SIR smaller 1 indicates a reduced risk.
3. Probability that area-specific SIR is >1: The probability that the region has an increased risk for KD compared to the rest of the study area. These exceedance probabilities were directly obtained from the corresponding posterior distributions ($P\left( \zeta_{i}>1 \right)$ = 1 – $P$ ($\zeta_{i}\leq1)$).

Note that one area in the Greater Toronto Area has no data (white spot) because there are no inhabitants (Toronto Pearson International Airport).

**Supplementary information on methods**

**Model formulation**

The number of children with KD in area $i$ and month $t$ (denoted by $Y_{it}$) is modelled as a Poisson distribution with SIR $\rho_{it}$ and the expected number of cases $E_{it}$ per person-year as

$$Y_{it}\sim\mathrm{Poisson}\left( {E_{it}\rho}_{it} \right).$$

$E_{it}$ is calculated by multiplying the at-risk population (i.e. the number of children <18 years old) times the overall KD incidence rate over the entire time period across all regions.

The linear predictor

$$\eta_{it} = ln \rho_{it}$$

can be further decomposed into spatial and temporal components. The spatial component is modelled following the standard Besag-York-Mollie (BYM) model^1^, which accounts for spatial autocorrelation. The BYM model includes a spatially unstructured $\upsilon_{i}$ and structured $u_{i}$ component. The unstructured random effect is independent mean-zero normally distributed with unknown variance. The spatially structured component is modelled as an intrinsic Gaussian Markov random field,^2^ and accounts for spatial autocorrelation. It essentially relates risk estimates to neighboring estimates and thereby smooths the effect across the area. Results are therefore dependent on how neighborhood is defined. Although distance-based neighborhood structures are a good choice if variations between sizes of the areas are large, we here choose adjacency-based structures because we are mainly interested in the relationship between Spatiotemporal covariates and KD. Boundary-based adjacency-matrices where a single shared boundary point meets the contiguity condition have been shown to result in less spatial smoothing, which is desirable to be able to detect the effect of covariates.^3^

We consider three temporal components in the model specification. The first component is a linear time trend $\phi_{t}$ and the second component a dynamic non-parametric trend $\gamma_{t}$ modelled through a random walk of first order (RW1), following Knorr-Held (2000).^4^ Due to the possible seasonal variations in KD, we also included a seasonal latent effect. In this case, the linear predictor can be written as

$\eta_{it}=u_{i}+\upsilon_{i}+\gamma_{t}+\phi_{t}+{seasonal}_{t}$.

**Model fitting**

Bayesian statistical conclusions about a parameter, $\theta$ are made in terms of probability statements that are conditional on the observed data $y$.

$$\begin{matrix} p\left( \theta| y \right)=\frac{p\left( y | \theta\right)p\left( \theta\right)}{p\left( y \right)}, \end{matrix}$$

describes the probability of an event based on prior information that might be related to that event. Since we do not assume conjugacy, we cannot obtain the posterior distribution analytically. A common approach is to use simulation methods (Markov Chain Monte Carlo), which are computationally intensive, especially in a Spatiotemporal setting with multiple covariates. We here use an alternative method, the integrated nested Laplace approximation (INLA) approach, which returns accurate parameter estimates in a short computation time, as was shown in Rue et al.^5^ We used a deterministic algorithm for Bayesian inference proposed by Rue et al.,^5^ made available through the R-INLA interface.^6^

The priors used for modelling the different effects are listed in Table 1.

Table 1: Choice of priors

| Model | Prior distribution | Shape | Rate |
| --- | --- | --- | --- |
| Spatial $\upsilon_{i}$ | Log-gamma | 1 | 0.00005 |
| Spatial $u_{i}$ | Log-gamma | 1 | 0.00005 |
| Linear time $\phi_{t}$ | Log-gamma | 1 | 0.00005 |
| Random walk I ($\gamma)$ | Log-gamma | 1 | 0.00005 |
| Seasonal | Log-gamma | 1 | 0.00005 |

The priors for the covariates were normally distributed with mean 0 and standard deviation 1000.

**Model selection and selection criterion**

The choice between different models was made based on the Watanabe-Akaike information criterion (WAIC). It estimates the out-of-sample expectation, and takes into account the effective number of parameters to adjust for overfitting. WAIC has been calculated following Gelman.^7^ It leads to similar results as leave-one-out cross-validation.^8^ In Bayesian statistics, the deviance information criterion (DIC) is often used for model selection. While the DIC is conditioned on point estimates, WAIC averages over the posterior distribution. This is generally advantageous in a Bayesian setting.

We assessed and compared models with different temporal structures: The first model had only the linear term $\gamma_{t}$, the second one $\gamma_{t}+\phi_{t}$, and the third one $\gamma_{t}+\phi_{t}+{seasonal}_{t}$. The latter model had the lowest WAIC and was therefore used for the main results of the study, which included covariates related to weather, pollution, and exposure to infections and biological particles. Variables to adjust for differences in incidences between different ethnicities and age-groups were first added to the model. We referred to that model as adjusted basemodel. Covariates were linearly added to the model, similar to the methods detailed by Fahrmeir and Lang.^9^

**References**

1. Besag, J., York, J. & Mollie, A. Bayesian Image-Restoration, with 2 Applications in Spatial Statistics. *Ann I Stat Math* 43, 1-20 (1991).

2. Besag, J. Spatial Interaction and the Statistical Analysis of Lattice Systems. *Journal of the Royal Statistical Society Series B (Methodological)* 36, 192-236 (1974).

3. Earnest, A.*, et al.* Evaluating the effect of neighbourhood weight matrices on smoothing properties of Conditional Autoregressive (CAR) models. *International journal of health geographics* 6, 54 (2007).

4. Knorr-Held, L. Bayesian modelling of inseparable space-time variation in disease risk. *Stat Med* 19, 2555-2567 (2000).

5. Rue, H., Martino, S. & Chopin, N. Approximate Bayesian inference for latent Gaussian models by using integrated nested Laplace approximations. *J R Stat Soc B* 71, 319-392 (2009).

6. Blangiardo, M. & Cameletti, M. Spatial and Spatio-temporal Bayesian Models with R-INLA Introduction. *Spatial and Spatio-Temporal Bayesian Models with R-Inla*, 1-18 (2015).

7. Gelman, A., Hwang, J. & Vehtari, A. Understanding predictive information criteria for Bayesian models. *Stat Comput* 24, 997-1016 (2014).

8. Watanabe, S. Asymptotic Equivalence of Bayes Cross Validation and Widely Applicable Information Criterion in Singular Learning Theory. *J Mach Learn Res* 11, 3571-3594 (2010).

9. Fahrmeir, L. & Lang, S. Bayesian inference for generalized additive mixed models based on Markov random field priors. *J Roy Stat Soc C-App* 50, 201-220 (2001).
